# Trends of Serum Electrolyte Changes in Crush syndrome patients of Bam Earthquake; a Cross sectional Study

**Published:** 2017-01-08

**Authors:** Saeed Safari, Mehdi Eshaghzade, Iraj Najafi, Alireza Baratloo, Behrooz Hashemi, Mohammad Mehdi Forouzanfar, Farhad Rahmati

**Affiliations:** 1 Emergency Department, Shohadaye Tajrish Hospital, Shahid Beheshti University of Medical Sciences, Teharn, Iran.; 2 Department of Nephrology, Dr. Shariati Hospital, Tehran University of Medical Sciences, Tehran, Iran.

**Keywords:** Earthquakes, crush syndrome, water-electrolyte imbalance, rhabdomyolysis, disaster victims

## Abstract

**Introduction:**

Electrolyte imbalances are very common among crushed earthquake victims but there is not enough data regarding their trend of changes. The present study was designed to evaluate the trend of changes in sodium, calcium, and phosphorus ions among crush syndrome patients.

**Methods:**

In this retrospective cross-sectional study, using the database of Bam earthquake victims, which was developed by Iranian Society of Nephrology following Bam earthquake, Iran, 2003, the 10-day trend of sodium, calcium, and phosphorus ions changes in > 15 years old crush syndrome patients was evaluated.

**Results:**

118 patients with the mean age of 25.6 ± 6.9 years were studied (57.3 male). On the first day of admission, 52.5% (95% CI: 42.7 - 62.3) of the patients had hyponatremia, which reached 43.9% (95% CI: 28.5 - 59.3) on day 10. 100.0% of patients were hypocalcemic on admission and serum calcium level did not change dramatically during the 10 days of hospitalization. The prevalence of hyperphosphatemia on the first day was 90.5% (95% CI: 81.5 - 99.5) and on the 10^th^ day of hospitalization 66.7% (95% CI: 48.5 - 84.8) of the patients were still affected.

**Conclusion:**

The results of the present study shows the 52.5% prevalence of hyponatremia, 100% hypocalcemia, and 90.5% hyperphosphatemia among crush syndrome patients of Bam earthquake victims on the first day of admission. Evaluation of 10-day trend shows a slow decreasing pattern of these imbalances as after 10 days, 43.9% still remain hyponatremic, 92.3% hypocalcemic, and 66.7% hypophosphatemic.

## Introduction

Natural disasters such as earthquake are unpredictable and unavoidable happenings that bring about considerable outcomes for the population health (1). These accidents may lead to immediate death by damaging vital organs (2). However, delayed death as a result of crush syndrome, which is the second most common cause of death after earthquakes behind trauma, is a common event among earthquake survivors (3). Crush syndrome happens following traumatic rhabdomyolysis, due to long-term and constant pressure on muscle bulks. Breakdown of rhabdomyocyte membranes lead to evacuation of cell contents in the blood flow and entrance of blood contents into the cell (4). This results in electrolyte imbalances such as hyperkalemia, myoglobinuria, hyperphosphatemia, hypocalcemia, etc. (5-7). Existing studies show that electrolyte imbalances are frequently seen in earthquake victims with crush syndrome; among which, hyperkalemia is the most dangerous one and causes a considerable proportion of deaths among these patients (8). The correlation of other electrolyte imbalances with mortality and unfavorable outcome in the injured has also been proved. However, there is little data regarding evaluation of electrolyte imbalances in crush syndrome patients and few studies are available in this regard (9-11). The reason might be found in the chaotic situation after disasters and lack of reliable data. 

The present study was designed aiming to evaluate the trend of electrolyte imbalances among Bam earthquake’s crush syndrome victims.

## Methods

***Study design and setting***

In the present cross-sectional study, data of Bam earthquake victims were retrospectively evaluated to assess the 10-day trend of changes in serum electrolyte levels including sodium, calcium, and phosphorous. 20-days trend of potassium changes was reported in another article, comprehensively. This study was approved by the Ethics Committee of Shahid Beheshti University of Medical Sciences, Tehran, Iran. During the study, researchers adhered to the principles of Helsinki Declaration and confidentiality of patient information.

***Participants***

Immediately after Bam earthquake in 2003 (Kerman province, southeastern Iran), a database was created based on questionnaires distributed by Iranian Society of Nephrology with the association of International Society of Nephrology in 7 major cities including 15 health centers. In this database, data of 4552 earthquake victims were recorded. Data included in this database were used to reach the aims of the present study. Details of data gathering and management have been described in previous studies (12-14). 

Patients that suffered from crush syndrome, whose serum electrolyte levels including sodium, potassium, calcium, and phosphorus were recorded for at least 3 days were included. Patients under 15 years of age, those who had chronic kidney diseases and patients whose creatine phosphokinase (CPK) level was never measured were excluded.

Crush syndrome is defined as a traumatic injury leading to a creatinine level more than 1.66 mg/dL and CPK over 1000 IU/L in at least 2 measurements during hospitalization (15). Sodium level under 135 mEq/L was considered hyponatremia and over 145 mEq/L hypernatremia. Hypokalemia was defined as serum potassium level under 3.5 mEq/L and levels over 5 mEq/L as hyperkalemia. In addition, calcium level over 10.2 mg/dL and lower than 8.7 mg/dL were defined as hypercalcemia and hypocalcemia, respectively. Normal phosphorus levels were also considered to be between 2.5 – 3.4 mg/dL (16).

***Statistical analysis***

Data were analyzed using STATA 11.0 statistical software. Serum level of each evaluated electrolyte was reported as mean ± standard deviation (SD). Afterwards, the prevalence of electrolyte imbalances including hyponatremia/hypernatremia, hypocalcemia/hypercalcemia, and hypophosphatemia/hyperphosphatemia were reported as percentage and 95%CI.

## Results

**Figure 1:**
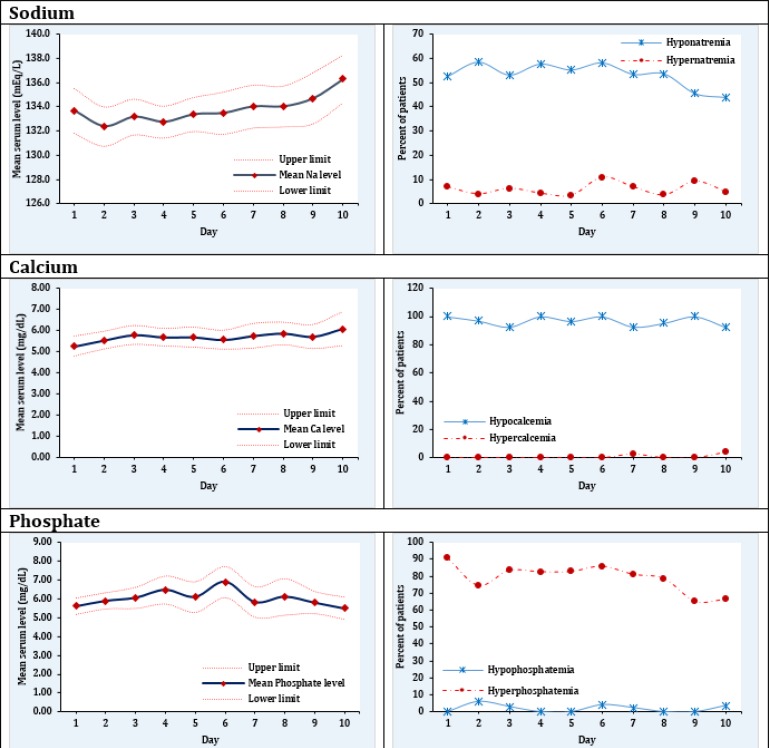
Mean and 95% confidence interval of serum sodium, calcium, and phosphate levels and their abnormalities during the initial 10 days after crush injury

Only 118 patients with the mean age 25.6 ± 6.9 years had documented electrolyte evaluation in the first 3 days of injury (57.3 male). Demographic, clinical and laboratory findings of these patients are presented in table 1. Mean and SD of sodium, potassium, calcium and phosphorus in the first 3 days were 133.8 ± 9.5 mEq/L, 5.7 ± 1.3 mEq/L, 5.2 ± 1.6 mEq/L and 6.2 ± 1.6 mEq/L, respectively. Trend of changes in mean level of the mentioned ions with 95% CI throughout 10 days of follow-up is shown in figure 1 and tables 2 to 4.

On the first day of admission, 52.5% (95% CI: 42.7 - 62.3) of the patients had hyponatremia and 6.9% (95% CI: 1.9 - 11.9) had hypernatremia (table 2). These rates were similar until the 8^th^ day of hospitalization. However, on the 9th and 10th days of follow-up the prevalence of hyponatremia decreased and reached 45.5% (95% CI: 30.5 - 60.4) and 43.9% (95% CI: 28.5 - 59.3), respectively (figure 1). 

Serum calcium level did not change dramatically during the 10 days of hospitalization. The interesting point was the 100.0% (95% CI: 99.0 - 100.0) prevalence of hypocalcemia on the day of admission. However, even after 10 days of hospital stay, hypocalcemia was detected in 92.3% (95% CI: 81.8 - 100.0) of the patients. On the 10^th^ day 3.8% (95% CI: 0.1 - 11.4) of the patients had hypercalcemia (table 3 and figure 1).

The prevalence of hyperphosphatemia on the first day was 90.5% (95% CI: 81.5 - 99.5) and no case of hypophosphatemia was present. Although the prevalence of hyperphosphatemia decreased as the days passed, on the 10^th^ day of hospitalization 66.7% (95% CI: 48.5 - 84.8) of the patients were still affected. Hypophosphatemia was seen in 3.7% (95% CI: 0.0 - 11.0) of the patients on the 10^th^ day (table 4 and figure 1).

**Table 1:** Baseline characteristics of included patients

**Variable**	**Value**
**Age (year)**	
15-24	104 (88.1)
25-64	6 (5.1)
≥65	8 (6.8)
**Time under the rubble (hour)**	6.2 ± 3.4
**Systolic blood pressure (mmHg)**	128.2 ± 21.1
**Diastolic blood pressure (mmHg)**	79.6 ± 12.1
**Fluid intake (ml)**	3147.0 ± 1998.0
**Urine output (ml)**	1167.0 ± 1186.0
**Blood urea nitrogen (mg/dL)**	102.0 ± 57.0
**Creatinine** **(mg/dL)**	4.6 ± 2.3
**Creatine phosphokinase** **(IU/L)**	19465 ±26078
**Lactate dehydrogenase** **(U/L)**	3331 ± 2518
**Uric acid** **(mg/dL)**	8.7 ± 2.9

Data were presented as mean ± standard deviation or number (%).

**Table 2:** Trend of serum sodium concentration during the initial 10 days after admission

**Day**	**Hyponatremia**		**Normal**		**Hypernatremia**	
	Prevalence	95% CI	Prevalence	95% CI	Prevalence	95% CI
1	52.5	42.7 - 62.3	40.6	31.0 - 50.2	6.9	1.9 - 11.9
2	58.4	48.7 - 68.1	37.6	28.1 - 47.1	4.0	0.1 - 7.8
3	53.1	43.1 - 63.2	40.6	30.7 - 50.5	6.3	1.4 - 11.1
4	57.6	47.4 - 67.8	38.0	28.1 - 48.0	4.3	0.2 - 8.5
5	55.2	44.6 - 65.7	41.4	31.0 - 51.8	3.4	0 - 7.3
6	58.1	46.8 - 69.4	31.1	20.4 - 41.7	10.8	3.7 - 17.9
7	53.4	40.5 - 66.4	39.7	26.9 - 52.4	6.9	0.3 - 13.5
8	53.6	40.4 - 66.8	42.9	29.8 - 56.0	3.6	0 - 8.5
9	45.5	30.5 - 60.4	45.5	30.5 - 60.4	9.1	0.5 - 17.7
10	43.9	28.5 - 59.3	51.2	35.7 - 66.7	4.9	0 - 11.6
**Total**	**53.7**	**50.2-57.2**	**40.3**	**36.8-43.8**	**6.0**	**5.3-7.7**

**Table 3:** Trend of serum calcium concentration during the initial 10 days after admission

**Day**	**Hypocalcemia**		**Normal**		**Hypercalcemia**	
	Prevalence	95% CI	Prevalence	95% CI	Prevalence	95% CI
1	100.0	99.0 - 100.0	3.0	0.1 - 7.1	0.0	0.0 - 0.0
2	97.0	100.0 - 100.0	7.4	1.1 - 13.6	0.0	0.0 - 0.0
3	92.6	86.4 - 98.9	0.0	0.0 - 0.0	0.0	0.0 - 0.0
4	100.0	99.0 - 100.0	3.3	0.1 - 7.8	0.0	0.0 - 0.0
5	96.7	92.2 - 100.0	0.0	0.0 - 0.0	0.0	0.0 - 0.0
6	100.0	99.0 - 100.0	4.9	0.1 - 11.6	0.0	0.0 - 0.0
7	92.7	100.0 - 100.0	4.7	0.1 - 11.0	2.4	0.1 - 7.2
8	95.3	100.0 - 100.0	0.0	0.0 - 0.0	0.0	0.0 - 0.0
9	100.0	99.0 - 100.0	3.8	0.1 - 11.4	0.0	0.0 - 0.0
10	92.3	81.8 - 100.0	3.0	0.1 - 7.1	3.8	0.1 - 11.4
**Total**	**96.6**	**95.1-98.2**	**3.0**	**1.5-4.4**	**0.4**	**0.0-0.0**

**Table 4:** Trend of serum phosphate concentration during the initial 10 days after admission

**Day**	**Hypophosphatemia**		**Normal**		**Hyperphosphatemia**	
	Prevalence	95% CI	Prevalence	95% CI	Prevalence	95% CI
1	0.0	0.0 – 0.0	9.5	0.5 – 18.5	90.5	81.5 – 99.5
2	6.0	0.2 – 11.7	19.4	9.8 – 29.0	74.6	64.1 – 85.2
3	3.0	0.0 – 7.2	13.6	5.3 – 22.0	83.3	74.3 – 92.4
4	0.0	0.0 – 0.0	17.5	7.6 – 27.5	82.5	72.5 – 92.4
5	0.0	0.0 – 0.0	17.3	6.9 – 27.7	82.7	72.3 – 93.1
6	4.1	0.0 – 9.7	10.2	1.6 – 18.8	85.7	75.8 – 95.6
7	2.4	0.0 – 7.1	16.7	5.2 – 28.1	81.0	68.9 – 93.0
8	0.0	0.0 – 0.0	21.4	8.8 – 34.0	78.6	66.0 – 91.2
9	0.0	0.0 – 0.0	34.5	16.8 – 52.1	65.5	47.9 – 83.2
10	3.7	0.0 – 11.0	29.6	12.0 – 47.2	66.7	48.5 – 84.8
**Total**	**2.1**	**0.8 – 3.3**	**17.6**	**14.2-21.0**	**80.3**	**76.8-83.9**

## Discussion

The results of the present study shows the 52.5% prevalence of hyponatremia, 100% hypocalcemia, and 90.5% hyperphosphatemia among crush syndrome patients of Bam earthquake victims on the first day of admission. Evaluation of 10-day trend shows a slow decreasing pattern of these imbalances as after 10 days, 43.9% still remain hyponatremic, 92.3% hypocalcemic, and 66.7% hypophosphatemic.

In a study by Zhang et al. that evaluated 180 victims of Wenchuan earthquake, prevalence of hyponatremia was reported to be 50.6%. They expressed that hyponatremia increases the odds of mortality in earthquake victims up to 5.74 times (9). However, in other studies the reported prevalence has varied from 14.5% to 75% (10, 11, 17, 18). These differences might be due to the small sample size in some studies and various settings of hyponatremia development. Apart from these differences, evaluation of the trends of hyponatremia in the present study showed that this electrolyte imbalance persisted in 43.9% of the victims even until the 10^th^ day. 

The reason for the high rate of hyponatremia may be found in the high volume of received fluid and its type. Since serum bicarbonate should be added to the received fluid to provide urine alkalization, physicians commonly prefer half saline due to fear of increased tonicity of fluids, which can lead to hyponatremia or stabilizing it. High volume of fluid needed to produce at least 1cc urine/kg/hour, which might reach 6 -10 liters per day, can be a reason for dilutional hyponatremia. However, the range of serum sodium in these hyponatremic patients is usually between 130 -135 and is mostly asymptomatic and benign.

In crush syndrome patients, hypocalcemia may occur for 2 reasons. As a result of variations in cell membrane permeability due to crush syndrome, calcium enters the cell and phosphorus leaves it. Phosphorus leaving the cell and combining with calcium leads to excretion of these ions. On the other hand, kidney injury may prevent the synthesizing the active form of vitamin D and intensify hypocalcemia (19). Since hypocalcemia, increases cardiotoxicity of hyperkalemia, correcting the serum concentration of this ion is of great importance (20). 

The interesting finding of this study is the 100% prevalence of hypocalcemia in the studied patients, although it has been asymptomatic in almost all of them. There might be 2 reasons for this high prevalence. First, the measurement reported has been of total calcium and might have yielded a different result if they were corrected based on protein level and free serum calcium was calculated. On the other hand, knowing that during 10 days only a small portion of hypocalcemia cases were corrected, patients might have suffered from underlying calcium deficiency. In addition, the role of vigorous fluid resuscitation, urine alkalization, and hyperphosphatemia (due to combining with calcium and sedimentation) should not be overlooked. 

Hyperphosphatemia is not life-threatening, however in acute phases it can lead to hypotension, hyperreflexia and even convulsion (19, 21). Findings of the present study showed that most victims of earthquake had hyperphosphatemia.

Of course about half of hyperphosphatemic cases following vigorous fluid therapy were corrected until the 10^th^ day of hospitalization. Almost none of hyperphosphatemia cases were symptomatized.

## .Limitation:

A small portion of the patients entered the study due to missing data, which is a common problem among similar studies, is an important limitation. For example in Wenchuan earthquake in China the serum sodium level of only 180 patients was available (9). On the other hand, the present study was multi-centric and therefore the difference in the management of the patients in various centers and not knowing the type and severity of trauma are among the most important limitations of this study.

Since information on management and outcome of crush syndrome patients is rare, the authors decided to do this study and publish the findings despite all the afore-mentioned limitations.

## Conclusion:

The results of the present study shows the 52.5% prevalence of hyponatremia, 100% hypocalcemia, and 90.5% hyperphosphatemia among crush syndrome patients of Bam earthquake victims on the first day of admission. Evaluation of 10-day trend shows a slow decreasing pattern of these imbalances as after 10 days, 43.9% still remain hyponatremic, 92.3% hypocalcemic, and 66.7% hypophosphatemic.
